# Cadmium effects on superoxide dismutase 1 in human cells revealed by NMR

**DOI:** 10.1016/j.redox.2019.101102

**Published:** 2019-01-08

**Authors:** Panagis Polykretis, Francesca Cencetti, Chiara Donati, Enrico Luchinat, Lucia Banci

**Affiliations:** a Magnetic Resonance Center - CERM, University of Florence, Via Luigi Sacconi 6, 50019 Sesto Fiorentino, Florence, Italy; b Department of Experimental and Clinical Biomedical Sciences “Mario Serio”, University of Florence, Viale Morgagni 50, 50134 Florence, Italy; c Department of Chemistry, University of Florence, Via della Lastruccia 3, 50019 Sesto Fiorentino, Florence, Italy

**Keywords:** Cadmium, Superoxide dismutase 1, In-cell NMR, Oxidative stress, Metallothionein

## Abstract

Cadmium is a toxic pollutant that in recent decades has become more widespread in the environment due to anthropogenic activities, significantly increasing the risk of exposure. Concurrently, a continually growing body of research has begun to enumerate the harmful effects that this heavy metal has on human health. Consequently, additional research is required to better understand the mechanism and effects of cadmium at the molecular level. The main mechanism of cadmium toxicity is based on the indirect induction of severe oxidative stress, through several processes that unbalance the anti-oxidant cellular defence system, including the displacement of metals such as zinc from its native binding sites. Such mechanism was thought to alter the *in vivo* enzymatic activity of SOD1, one of the main antioxidant proteins of many tissues, including the central nervous system. SOD1 misfolding and aggregation is correlated with cytotoxicity in neurodegenerative diseases such as amyotrophic lateral sclerosis. We assessed the effect of cadmium on SOD1 folding and maturation pathway directly in human cells through in-cell NMR. Cadmium does not directly bind intracellular SOD1, instead causes the formation of its intramolecular disulfide bond in the zinc-bound form. Metallothionein overexpression is strongly induced by cadmium, reaching NMR-detectable levels. The intracellular availability of zinc modulates both SOD1 oxidation and metallothionein overexpression, strengthening the notion that zinc-loaded metallothioneins help maintaining the redox balance under cadmium-induced acute stress.

## Highlights

• Cadmium does not bind to superoxide dismutase 1 (SOD1) in human cells.
• In defect of zinc, cadmium causes the premature oxidation of SOD1.
• Cadmium induces the overexpression of metallothioneins to levels detectable by NMR.
• Zinc modulates metallothionein expression and attenuates SOD1 oxidation.

## Graphical abstract

**Figure:**
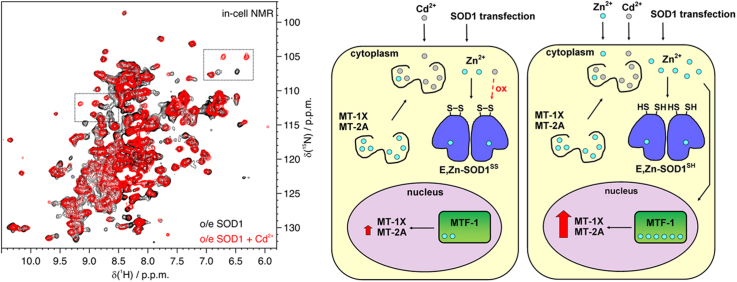

## 1 Introduction

Cadmium is a potent environmental and food contaminant that is ranked as the seventh most hazardous substance for human health when considering both toxicity and exposure frequency [1]. Furthermore, it has been classified as a category 1 carcinogen by the International Agency for Research on Cancer [2]. Cadmium can spread in the environment due to natural processes like volcanic eruptions and erosion, but during recent decades anthropogenic activities such as agriculture and industry have significantly boosted its dispersal in soil and water [3]. Cadmium compounds are very soluble in water, facilitating its uptake by plants and consequently human exposure as a food and tobacco contaminant [4], [5]. Cadmium is not a physiological element for any living organism, and furthermore is a cumulative toxin with an extremely long estimated half-life (up to 13.6–23.5 years) due to its very low excretion rate [6]. In mammals, the main accumulation sites of cadmium are kidneys, liver, lungs, bone, testes and brain, where it causes severe oxidative stress and other detrimental effects [7], [8]. Unlike iron and copper, cadmium is not a Fenton-like metal, consequently it is not involved directly in the production of Reactive Oxygen Species (ROS). Rather, it increases their intracellular production and their harmful effects by altering the balance of the cellular anti-oxidative defence system. In fact, cadmium is thought to be responsible for the replacement of metals from their catalytic sites [9], [10], [11], [12]; the depletion of anti-oxidant metabolites, like glutathione, ascorbic acid and vitamin E; the inhibition of the electron transport chain, resulting in mitochondrial damage; and the alteration of the enzymatic activity of anti-oxidant proteins [13], [14], [15]. One of the main cellular defence tools against cadmium are metallothioneins (MTs), a family of highly conserved, cysteine-rich small proteins with several key functions: toxic metal and radical scavenging, maintenance of the intracellular zinc homeostasis, maintenance of the intracellular redox balance and protection against DNA damage [16], [17], [18]. Mammalian MTs have about 60 amino acids, one third of which are cysteines, forming two metal-thiolate clusters. The protein fold is induced by metal ions (such as Zn^2+^, Cd^2+^ and Cu^+^), and cluster formation [19]. Human MTs have 11 functional isoforms divided in 4 subfamilies (MT1-4), which share a high degree of homology and are differentially expressed depending on the tissue [20]. The expression of MTs is known to be induced by Cd^2+^ exposure through a mechanism mediated by the release of intracellular free Zn^2+^ [21].

Among the anti-oxidant proteins affected by cadmium intoxication, literature reports the involvement of cadmium in the alteration of the enzymatic activity of superoxide dismutase 1 (SOD1) [13]. SOD1 is a metalloprotein that exerts a fundamental anti-oxidant role catalysing the dismutation of O_2_^•-^ to H_2_O_2_ and O_2_ with extremely high reaction rates [22], [23], [24]. SOD1 enzymatic activity is essential for the maintenance of the physiological cellular conditions and requires correct protein folding, metal binding and disulfide bond formation. The impairment of SOD1 maturation has been also related to the onset of severe disease states, including amyotrophic lateral sclerosis (ALS) [25], [26], [27], [28], [29].

*In vitro*, cadmium has been shown to bind to the zinc and copper sites of SOD1 [30], which are necessary for the structural stability and catalytic activity of SOD1, respectively. *In vivo*, however, opposing effects have been reported on SOD1 activity in response to cadmium exposure, and evidence of alteration at the protein level is lacking [31], [32], [33]. Therefore, it is not clear whether/how cadmium affects SOD1 folding and metallation within the cell, *i.e.* in the presence of the native SOD1 metal ions and under the control of the cellular metal and redox homeostasis.

Given these contrasting premises, we sought to evaluate the effects of cadmium treatment on the maturation of SOD1 in human cells by in-cell NMR, to determine whether cadmium binds to the zinc and/or copper sites or it affects intracellular SOD1 maturation by other mechanisms. To this aim, in-cell NMR is the ideal technique, as it is able to analyse proteins at atomic resolution directly in living cells. The same technique has been applied previously to observe changes in the intracellular SOD1 folding, metallation and redox state as a consequence of the physiological maturation and/or in response to external stimuli [34], [35], [36], [37].

## 2 Materials and methods

### 2.1 In-cell NMR

In-cell NMR experiments have been performed as previously described [38] on living human embryonic kidney cells (HEK293T), under three main experimental conditions: i) exposure to Zn^2+^ (added in the culture at the time of transfection with SOD1); ii) exposure to Cd^2+^ (added in the culture 24 h after the transfection with SOD1); iii) exposure to Zn^2+^and Cd^2+^ (both added in the culture at the aforementioned times). HEK293T cells were grown on uncoated 75 cm^2^ plastic flasks at 37 °C in 5% CO_2_ atmosphere, and were maintained in Dulbecco's Modified Eagle's medium (DMEM; high glucose, D6546, Sigma-Aldrich, St. Louis, MO) supplemented with L-glutamine, antibiotics (penicillin and streptomycin) and 10% foetal bovine serum (FBS) (Gibco-Thermo Fisher Scientific, Waltham, MA). Cells were transiently transfected with the pHLsec plasmid [39] encoding for human SOD1, using polyethylenimine (PEI) in the ratio 1:1 (25 μg each), in ^15^N labelled media (BioExpress6000, Cambridge Isotope Laboratories, Inc., Tewksbury, MA), supplemented 2% FBS in the presence/absence of Zn^2+^ as ZnSO_4_ 10 μM. Under these conditions, ~150 μM SOD1 is expressed [38]. To decrease the expression levels of SOD1, the pHLsec encoding SOD1 was mixed 1:1 with empty vector and transfected as above, resulting in the expression of ~65 μM SOD1. 24 h after the transfection, 10 μM of CdCl_2_ was added to the cell cultures; such concentration was chosen considering previous experiments performed on Hep3B and N2A cells [32], [40]. After 24 h of exposure to cadmium, the cells were washed twice with PBS, trypsinised, spun at 500 g after trypsin inactivation, resuspended once in PBS and spun down again at 500 g. Such procedure allows efficient removal of debris from dead cells and of apoptotic cells, if present. Cell viability was assessed both before and after NMR analysis by counting cells stained with trypan blue using a Burker chamber. Cd^2+^ treatment caused a reduction of ~40% in the final number of cells analysed by NMR, likely due to cell death/apoptosis. However, the fraction of cells treated with Cd^2+^ that was recovered and analysed by NMR had the same viability as the Cd^2+^-untreated cells (>95% trypan blue-negative before the NMR experiments, >90% after the NMR experiments). For NMR analysis, the recovered cells were collected and placed in a 3 mm Shigemi NMR tube. ^1^H WATERGATE (3-9-19) and ^1^H–^15^N SOFAST-HMQC NMR spectra were acquired on living HEK293T cells and on lysates at a 950 MHz Bruker (Billerica, MA) Avance III or at a 900 MHz Bruker Avance HD spectrometer both equipped with a TCI CryoProbe, at 308 K. The cell lysates were obtained by freeze-thaw lysis in phosphate buffered saline (PBS) buffer, pH 7.4, followed by centrifugation at 14,000 rpm. For the [^15^N]-cysteine selective labelling of MTs, untransfected HEK293T cells were grown in homemade medium containing [^15^N]-cysteine (Cambridge Isotope Laboratories, Inc.); NMR spectra were acquired on the corresponding cell lysate at 298 K. All NMR spectra were acquired and processed using Bruker Topspin software. The uniformly-^15^N labelled in-cell NMR spectra were further processed by subtracting a spectrum of cells transfected with empty vector, acquired in the same experimental conditions, to eliminate the signals arising from partial ^15^N labelling of other cellular components.

The intracellular oxidation state of E,Zn-SOD1 was determined from the intensities of the signals arising from G10 and N53 Nδ1 in each oxidation state in the ^1^H–^15^N NMR spectra. The amount of oxidised/reduced E,Zn-SOD1 in each condition are reported as percentages over total E,Zn-SOD1 calculated for two independent cell samples.

### 2.2 In vitro NMR

Recombinant SOD1 was demetallated as previously described [41] by repeated dialyses against 10 mM EDTA in 50 mM acetic acid at pH 3.5. The buffer was then replaced with PBS, pH 7.4. Reduction of SOD1 disulfide bond was performed by incubating apo-SOD1^SS^ with 50 mM of DTT for 40 min at 37 °C. DTT was then removed in oxygen-free PBS buffer.

*In vitro* NMR titration experiments were performed at 950 MHz in anaerobic conditions. E,Zn-SOD1^SH^ was obtained by titrating 150 μM apo-SOD1^SH^ in PBS buffer, pH 7.4, with 1 equivalent of ZnCl_2_. Titration with Cd^2+^ was performed by addition of increasing equivalents of CdCl_2_ to a sample of 150 μM apo-SOD1^SH^. ^1^H WATERGATE (3-9-19) and ^1^H–^15^N SOFAST-HMQC NMR spectra were acquired at 298 K after each addition. Disulfide-oxidised E,Zn-SOD1 was prepared by titration of apo-SOD1^SS^ with one equivalent of Zn^2+^ monitored *via* NMR.

### 2.3 Real-time PCR

Gene expression analysis by Real-Time PCR has been performed using the 2^(-ΔΔCT) comparative method of quantification [42]. Briefly, total RNA (1 µg), extracted with TriReagent™ (Sigma-Aldrich) was reverse transcribed using iScript™ cDNA Synthesis Kit for RT-qPCR (Bio-Rad Laboratories, Hercules, CA) according to the manufacturer's instructions. Human specific TaqMan Gene Expression Assays employed for gene expression studies were purchased from Thermo Fisher Scientific and listed in Table S1. The quantification of target gene mRNA levels was performed employing TaqMan Universal Master Mix (Thermo Fisher Scientific) with the automated ABI Prism 7500 Sequence Detector System (Thermo Fisher Scientific) as described previously [43]. Each measurement was carried out in triplicate, by analysing three independent cell samples. The quantification was performed by simultaneous amplification of the target gene together with the housekeeping (GAPDH) in order to normalise expression data. Results were analysed by ABI Prism Sequence Detection Systems software, version 1.7 (Applied Biosystems, Foster City, CA) and graphical representations of data as mean ± SD of three independent experiment were obtained by GraphPad Prism 5.0 (GraphPad Software, San Diego, CA).

## 3 Results

**Fig. 1:**
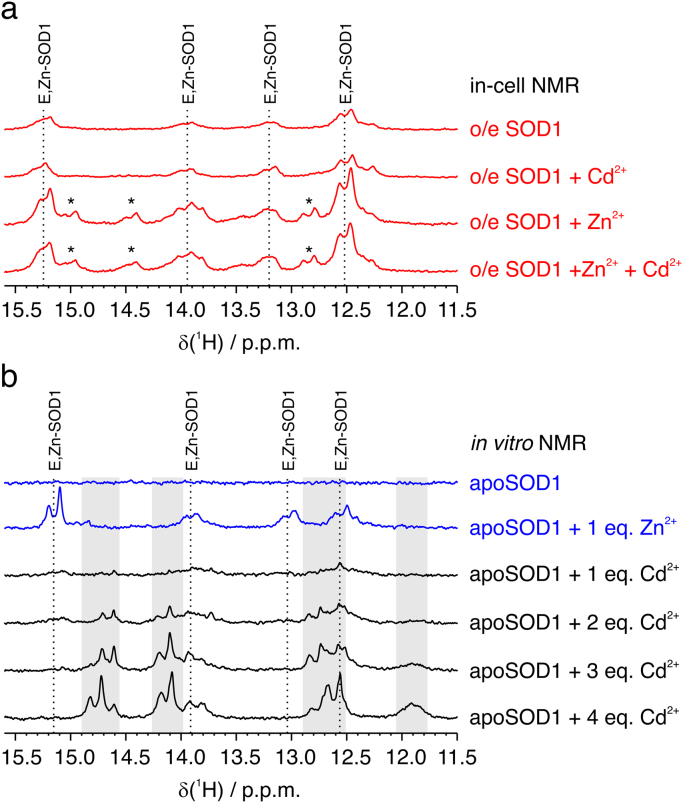
**Intracellular SOD1 binds Zn**^**2+**^**, not Cd**^**2+**^. Imino region of (a) in-cell ^1^H NMR spectra of cells expressing [U-^15^N] labelled SOD1 and treated with different metals (red); (b) *in vitro*^1^H NMR spectra of disulfide-reduced apo-SOD1 (120 μM) alone (blue), titrated with 1 equivalent per monomer of Zn^2+^ (blue) and titrated with 2, 3 and 4 equivalents per monomer of Cd^2+^ (black). The histidine signals corresponding to the E,Zn-SOD1 form are shown with dashed lines and labelled. Signals likely arising from the non-native Zn,Zn-SOD1 form are labelled with asterisks. Spectral regions containing signals arising from Cd^2+^ binding to SOD1 are shown in grey.

SOD1 overexpressed in human cells was analysed by in-cell NMR to determine its metallation and redox state under different conditions, by comparing the in-cell spectra with *in vitro* spectra of the protein in known states. The metallation state was determined from the chemical shift of the metal binding histidines in imino region of the ^1^H NMR spectra. Under basal conditions (*i.e.* without addition of metals), a fraction of overexpressed SOD1 binds the Zn^2+^ available in the growth medium while the remaining fraction is in the apo state (apo-SOD1, which gives no signals in the imino region), whereas addition of Zn^2+^ allows the complete Zn^2+^ binding to SOD1 with one Zn^2+^ per monomer (E,Zn-SOD1), as previously reported [34] (Fig. 1a). Exposure to Cd^2+^ for 24 h did not cause any Cd^2+^ binding to SOD1, as determined by comparison with the *in vitro* NMR spectra. Indeed, the histidine signals of intracellular SOD1 in the Cd^2+^-treated cells did not change with respect to the untreated cells, matched those of apo-SOD1 metallated with Zn^2+^ *in vitro* and differed from those of apo-SOD1 metallated *in vitro* with increasing equivalents of Cd^2+^ per SOD1 monomer (Fig. 1b). Moreover, the titration performed *in vitro* denotes that Cd^2+^ binds to SOD1 with lower affinity than Zn^2+^, as two equivalents or more of Cd^2+^ are necessary to observe spectral changes, suggesting that Cd^2+^ binding to apo-SOD1 or replacement of Zn^2+^ with Cd^2+^ is unlikely to occur in the cells.

**Fig. 2:**
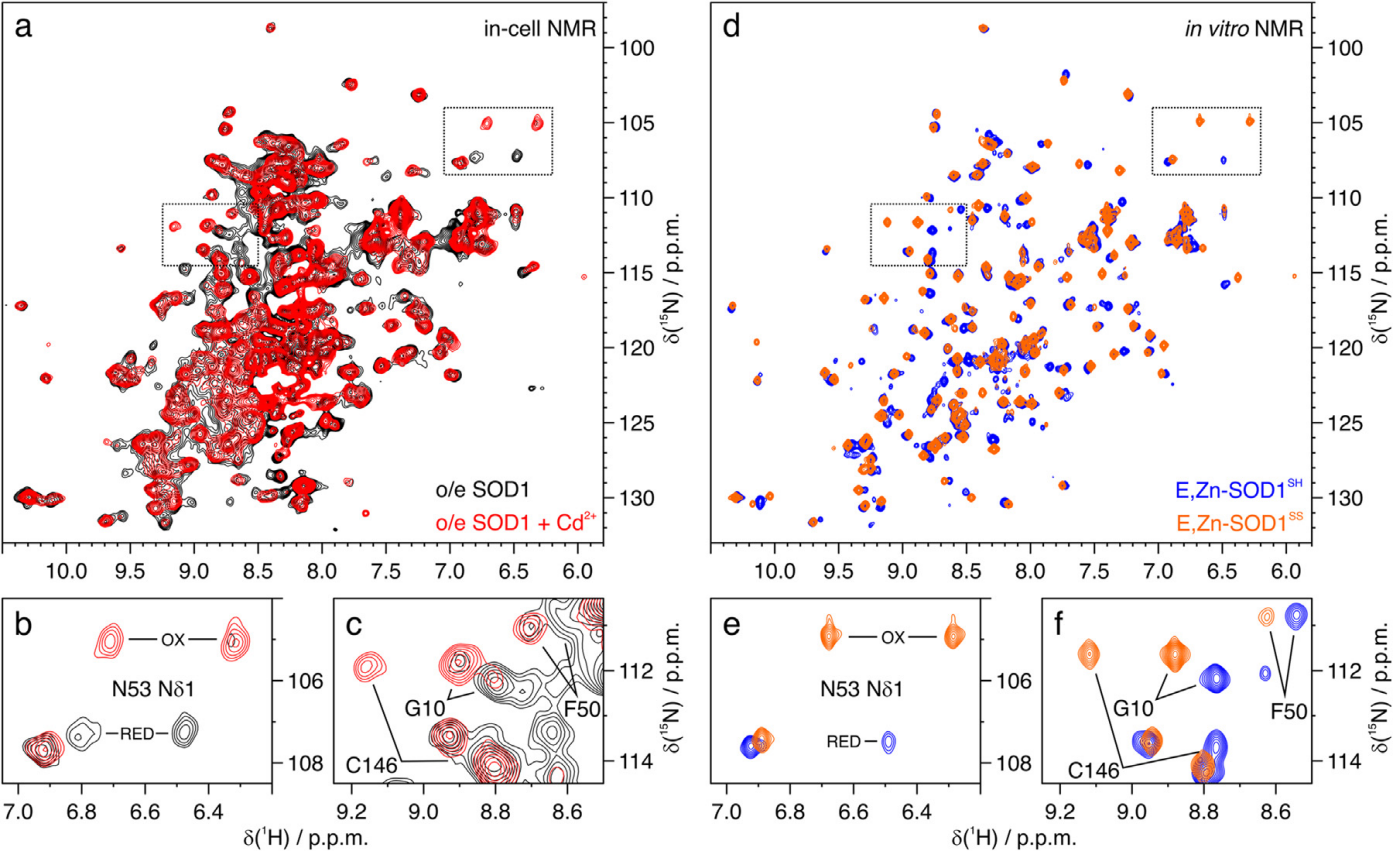
**Cd**^**2+**^**induces intracellular SOD1 oxidation**. (a) Overlay of in-cell ^1^H–^15^N NMR spectra acquired on cells expressing [U-^15^N] labelled SOD1 in defect of Zn^2+^ either untreated (black) or treated (red) with Cd^2+^; (d) overlay of *in vitro*^1^H–^15^N NMR spectra acquired on disulfide-reduced E,Zn-SOD1 (blue) and disulfide—oxidised E,Zn-SOD1 (orange); (b, c, e, f) enlarged areas of the same spectra (dotted rectangles in a, c). Representative signals affected by the formation of the C57-C146 disulfide bond are labelled.

Instead, Cd^2+^ treatment caused the oxidation of a sizable fraction of intracellular E-Zn,SOD1, *i.e.* the formation of an intramolecular disulfide bond between C57 and C146. In defect of Zn^2+^, the metal-free protein is fully disulfide-reduced (apo-SOD1^SH^), while the form with one Zn^2+^ bound per monomer is mostly in the reduced state (60–70% E,Zn-SOD1^SH^ over total E,Zn-SOD1, Fig. 2a). When Cd^2+^ was added to the cells in defect of Zn^2+^, a sizable fraction (65–75%) of the zinc-bound form was oxidised (E,Zn-SOD1^SS^), as revealed by the changes in relative signal intensity in the 2D NMR spectra, while the apo form was unaffected, *i.e.* it showed a NMR spectrum superimposable to that of the untreated sample, indicating that it is in the disulfide-reduced state (apo-SOD1^SH^) (Fig. 2a). The in-cell spectral changes induced by Cd^2+^ matched those observed *in vitro* between the reduced and oxidised forms, *i.e.* E,Zn-SOD1^SH^ and E,Zn-SOD1^SS^ (Fig. 2b). Notably, the same effect was observed in cells expressing SOD1 at lower levels, where Cd^2+^ caused the oxidation of ~63% of E,Zn-SOD1 (Fig. S1).

**Fig. 3:**
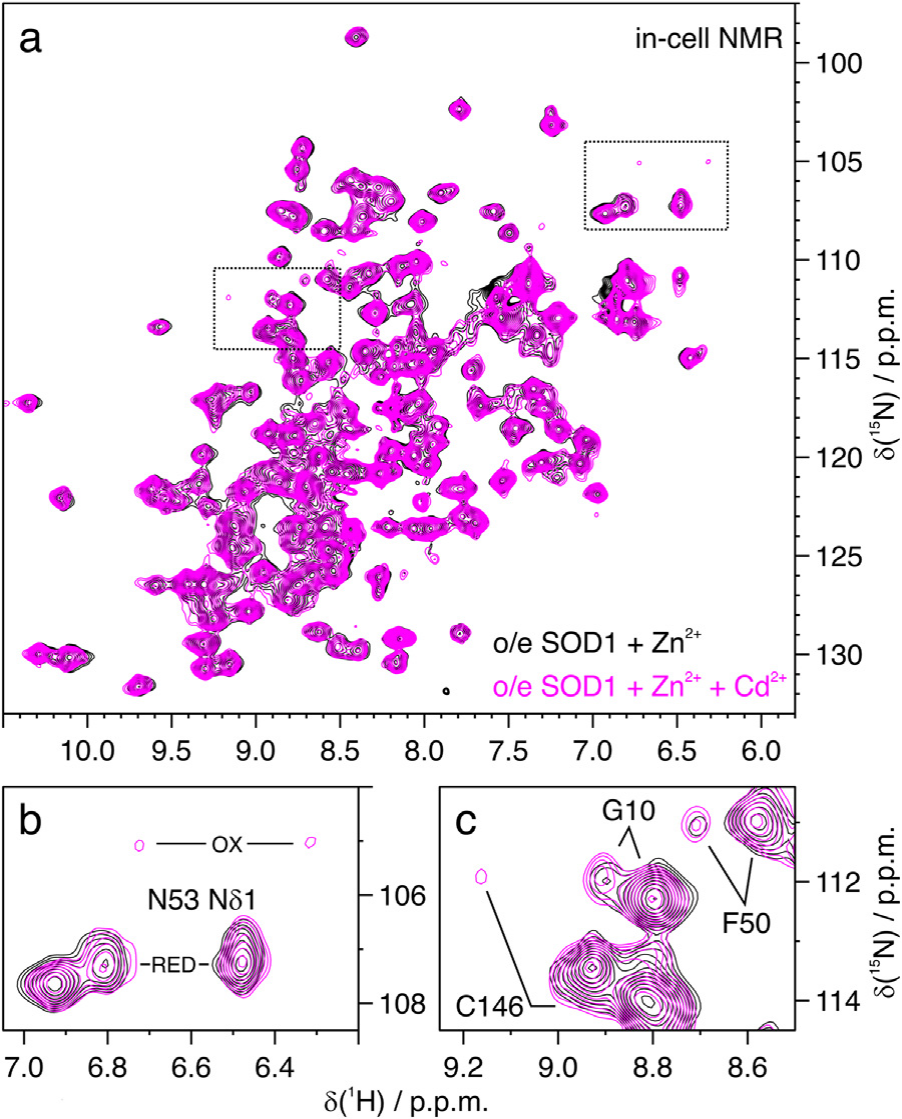
**Excess of Zn**^**2+**^**prevents Cd**^**2+**^**-induced SOD1 oxidation.** (a) Overlay of in-cell ^1^H–^15^N NMR spectra acquired on cells expressing [U-^15^N] labelled SOD1 in excess of Zn^2+^ either untreated (black) or treated (magenta) with Cd^2+^; (b, c) enlarged areas of the same spectra (dotted rectangles in a). Representative signals affected by the formation of the C57-C146 disulfide bond are labelled.

**Fig. 4:**
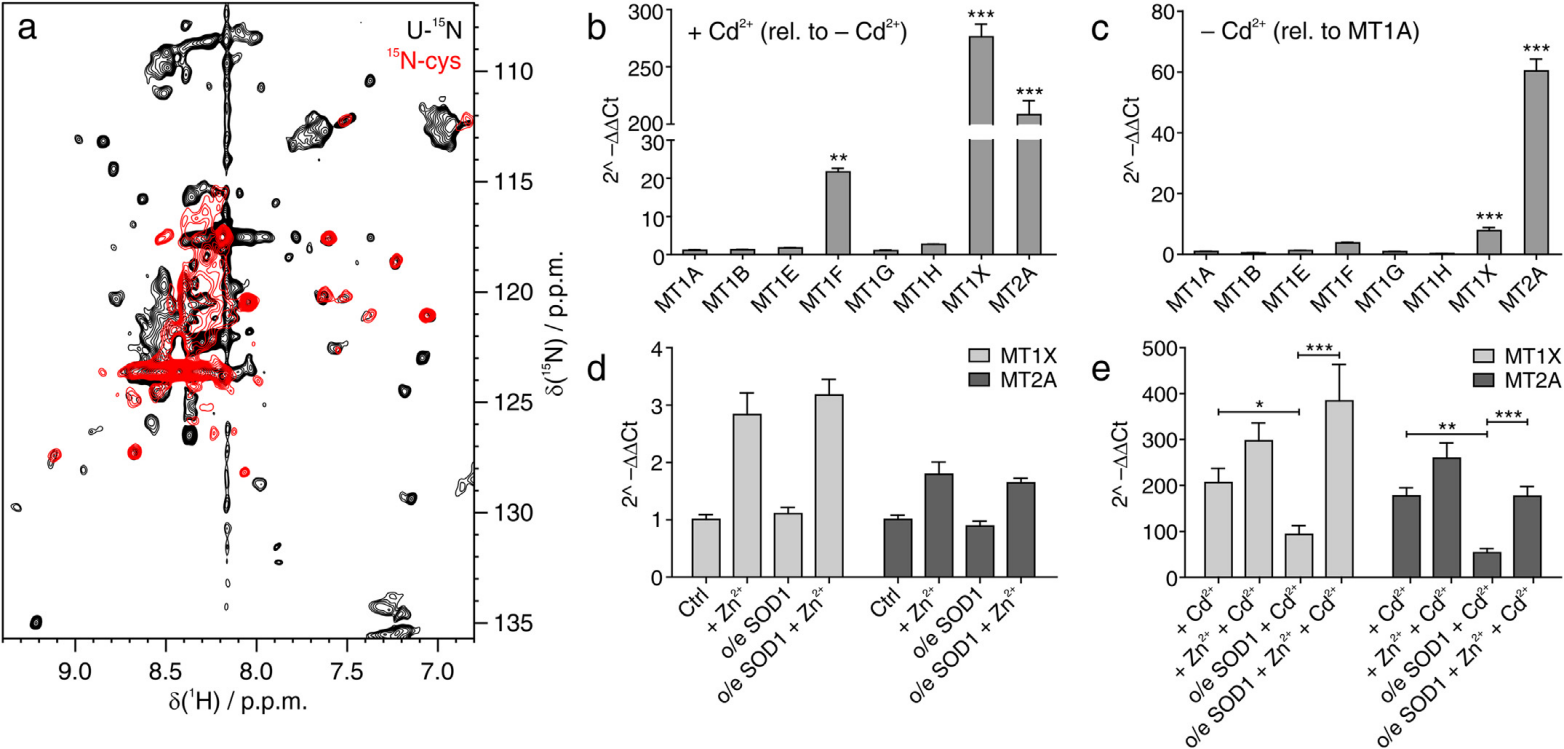
**Cd**^**2+**^**treatment induces MT overexpression to NMR-detectable levels.** (a) Overlay of ^1^H–^15^N NMR spectra acquired on untransfected cells treated with Cd^2+^ either [U-^15^N] labelled (black) or [^15^N-cysteine] labelled (red); (b–e) Real-Time PCR analysis: (b) expression of different MT isoforms in Cd^2+^-treated untransfected cells, relative to basal (*i.e.* untransfected cells not treated with Cd^2+^). Significant values are marked with asterisks (Student's *t*-test, ** = P < 0.01; *** = P < 0.001). (c) Basal expression of different MT isoforms relative to that of MT1A. Significant values are marked with asterisks (1-way ANOVA followed by Bonferroni post-hoc test, *** = P < 0.001). (d) Expression of MT1X and MT2A in cells not treated with Cd^2+^, overexpressing SOD1 in defect/excess of Zn^2+^, relative to basal. (e) Expression of MT1X and MT2A in cells treated with Cd^2+^, overexpressing SOD1 in defect/excess of Zn^2+^, relative to basal. Significant differences are marked with asterisks (2-way ANOVA followed by Bonferroni post-hoc test, * = P < 0.05, ** = P < 0.01, *** = P < 0.001). Ctrl: untransfected, untreated cells; +Zn^2+^: cells treated with excess Zn^2+^; +Cd^2+^: cells treated with Cd^2+^; o/e SOD1: cells overexpressing SOD1.

The oxidation of SOD1 induced by Cd^2+^ was largely attenuated by the addition of Zn^2+^. In the presence of excess Zn^2+^, intracellular E,Zn-SOD1 was mostly found in the reduced state (80–90% E,Zn-SOD1^SH^), as previously reported [34], [36]. When both Zn^2+^and Cd^2+^ were supplemented, a smaller fraction (25–45%) of SOD1 was oxidised (E,Zn-SOD^SS^), while the rest of the protein remained reduced, as that observed in the Zn^2+^-treated cell sample (Fig. 3). The considerably smaller fraction of oxidised SOD1, compared to the sample supplemented only with Cd^2+^, suggested that a protective mechanism against oxidative stress had been activated by supplementing Zn^2+^. We hypothesised that such effect could have been exerted by MTs, considering their protective role against cadmium, their involvement in zinc homeostasis and their intracellular anti-oxidant action. Indeed, MTs are well known to be overexpressed in cell lines and tissues as a consequence of cadmium exposure [44], [45]. Interestingly, analysing the NMR data, several additional amide crosspeaks not originating from any known state of SOD1 were observed in the 2D spectra of cells treated with both Cd^2+^ and Zn^2+^ (Fig. S2). To further investigate the origin of these signals, we performed additional in-cell NMR experiments on cells not transfected with the plasmid encoding for SOD1, that were grown in either uniform-^15^N or [^15^N]-cysteine labelled medium and subsequently exposed to Cd^2+^. The unknown crosspeaks were also present in the resulting NMR spectra (Fig. 4a), indicating that they did not arise from SOD1 but from some other protein(s) upregulated upon Cd^2+^ treatment. Strikingly, many signals (~20) were also present in the [^15^N]-cysteine labelled sample (Fig. 4a and Fig. S3). Such a high ratio of cysteine residues is typical of MTs. Therefore, MT overexpression could explain the additional crosspeaks in Cd^2+^-treated cells, and the lower amount of oxidised SOD1.

To confirm that MTs are overexpressed as a consequence of cadmium exposure and to identify which MT isoforms are upregulated the most, we performed Real-Time PCR experiments on mRNA extracts from cells either untreated or treated with Cd^2+^ and/or Zn^2+^. Real-Time PCR analysis revealed that, indeed, expression of MTs was greatly increased upon Cd^2+^ treatment in our experimental conditions (Fig. 4b and Table S2). Specifically, in cells treated with Cd^2+^ MT-1X expression was increased ~280-fold compared to the untreated cell sample, while MT-2A expression was increased ~210-fold; MT-1F was also overexpressed to a lesser extent (~20-fold). Considering that in the control sample MT-1X and MT-2A are expressed respectively ~8 and ~60 times more than MT-1A (taken as reference, Fig. 4c and Table S2), the levels of MT-1X and MT-2A in Cd^2+^-treated cells reached up to ~2200 and ~12500 times the basal MT-1A levels, respectively, by far the highest isoforms, thus suggesting that the observed NMR signals arise from these isoforms. In cells treated with both Cd^2+^ and Zn^2+^, an increase in the levels of MT-1X and MT-2A was observed (respectively, ~300- and ~260-fold), while in cells treated only with Zn^2+^ a much lower MT overexpression was observed (Fig. 4d,e and Table S3).

Real-Time PCR MTs expression analysis was also performed on cells overexpressing SOD1 as it occurs in the in-cell NMR samples upon supplementation of Cd^2+^ and/or Zn^2+^. Interestingly, in cells expressing SOD1 a different behaviour was observed in defect of Zn^2+^ *versus* excess of Zn^2+^, upon Cd^2+^ treatment. In cells expressing SOD1 in defect of Zn^2+^, Cd^2+^ treatment still induced the expression of MT-1X and MT-2A, but at significantly lower levels (~90- and ~50-fold, respectively, compared to basal levels) than in untransfected cells, while excess Zn^2+^ in SOD1-expressing cells significantly restored the Cd^2+^-induced expression of MT-1X and MT-2A to levels (~380- and ~180-fold, respectively) similar to those reached in untransfected cells (Fig. 4e and Table S3). These data are consistent with the fact that MTs were not detected by NMR in cells expressing SOD1 in defect of Zn^2+^ (Fig. S4).

Upregulation of MTs was much lower in cells (both untransfected and transfected with SOD1) treated with Zn^2+^ in excess but not exposed to Cd^2+^ (Fig. 4d). Taken together, these data indicate that cadmium is the main trigger of MT overexpression and that SOD1 and zinc supplementation can modulate Cd^2+^-induced MT overexpression in opposite ways.

## 4 Discussion

In-cell NMR allows a direct assessment of the intracellular state of SOD1 upon Cd^2+^ treatment, and therefore can provide a close-to-physiological picture of the effect of cadmium exposure on SOD1 in human cells. Previous *in vitro* NMR studies demonstrated that Cd^2+^ is able to bind to the zinc and copper sites of SOD1 [30], while studies on animal models showed that Cd^2+^ affects the superoxide dismutase activity in several tissues [31], [32], [33]. We therefore sought to determine whether direct binding of Cd^2+^ to SOD1 can occur in living cells. By in-cell NMR, we did not observe any direct Cd^2+^ binding to SOD1, as SOD1 was present only with one Zn^2+^ bound per monomer or in the apo state. Indeed, the chemical shifts of the signals arising from the metal-coordinating histidines observed in the Cd^2+^-treated cells were identical to those of E,Zn-SOD1 in-cell and *in vitro*, and differed from those observed when apo-SOD1 was titrated *in vitro* with increasing equivalents of Cd^2+^ (Fig. 1). Moreover, different species were simultaneously present during the titration and residual apo-SOD1 was still present after up to two equivalents of Cd^2+^. This behaviour is consistent with Cd^2+^ binding with low selectivity to either the zinc or the copper site of SOD1. At a protein concentration of 120 µM, the K_d_ can be estimated in the order of ~10^-4^ M, strikingly higher than that of Zn^2+^ binding to its native site (K_d_ = 4.2 ×10^-14^ M [46]), indicating that Cd^2+^ binding to intracellular SOD1 in place of Zn^2+^ would not be thermodynamically favoured.

Despite the lack of direct binding to intracellular SOD1, a clear effect of Cd^2+^ treatment was the oxidation of SOD1 cysteines, *i.e.* the formation of the intrasubunit disulfide bond between C57 and C146. This outcome is likely a consequence of an intracellular redox unbalance caused by Cd^2+^. The disulfide bond formation is required for the correct maturation of SOD1, as it stabilises the protein structure and is essential for its activity [47], [48]. Moreover, the ability of an exogenous redox-reactive compound to stabilise SOD1 by promoting disulfide oxidation has also been reported [37]. While cysteine oxidation contributes to stabilise the protein, it could interfere with the native maturation pathway, as SOD1 needs to interact with its partner copper chaperone for SOD1 (CCS) in order to bind copper [49], [50]. SOD1 interacts with CCS more efficiently in the reduced state [51], [52]. Upon interaction, CCS delivers copper to SOD1 and catalyses its oxidation, thus producing mature SOD1 [53], [54]. The premature oxidation of SOD1 could therefore prevent the interaction with CCS and impair copper delivery, as shown previously in yeast [55] and with human SOD1 *in vitro* [54]. Such effect would be consistent with the decrease of superoxide dismutase activity reported *in vivo* [13], [31]. Cd^2+^-mediated SOD1 oxidation did not occur in the presence of excess Zn^2+^, thus suggesting that Zn^2+^ could modulate the extent of the effect of Cd^2+^. Notably, this mechanism cannot involve mature Cu,Zn-SOD1, as the disulfide bond is already formed and Cd^2+^ would not be able to replace either zinc or copper, which is bound to SOD1 with even higher affinity.

Furthermore, we observed that Cd^2+^ treatment strongly induced the overexpression of the metallothionein isoforms MT-1X and MT-2A. Interestingly, while MT overexpression is a well known consequence of Cd^2+^ exposure [44], [45], MT-1X and MT-2A reached such high levels in our experimental conditions that they could be observed by in-cell NMR. Based on the estimated detection limit of the technique of ~5 µM for small folded proteins and on previous in-cell NMR analyses of other proteins [36], the concentration of MTs in the NMR tube was estimated around 5–10 µM. While in principle the high spectral resolution should allow separating the signals of MT-1X and MT-2A, only one set of resonances could be identified due to the low signal-to-noise ratio and likely because most signals have the same chemical shift due to the high structural similarity of the two isoforms, which have an overall sequence identity > 90% and share a central stretch of 38 identical residues. Strong MT induction required the presence of Cd^2+^, and was also observed in HEK293T not transfected with the SOD1 gene, where Zn^2+^ supplementation further potentiated the MT induction. Interestingly, SOD1 overexpression negatively modulated the level of Cd^2+^-mediated MT induction, and indeed in such experimental conditions, where SOD1 was partially oxidised upon Cd^2+^ treatment, MT-1X/MT-2A did not reach sufficiently high levels to be detected in the NMR spectrum. Conversely, supplementation of both Cd^2+^ and Zn^2+^ to cells overexpressing SOD1, *i.e.* in the experimental conditions where SOD1 was mostly reduced, Cd^2+^-mediated MT induction was restored, and high MT-1X/MT-2A levels were observed in the NMR spectrum.

**Fig. 5:**
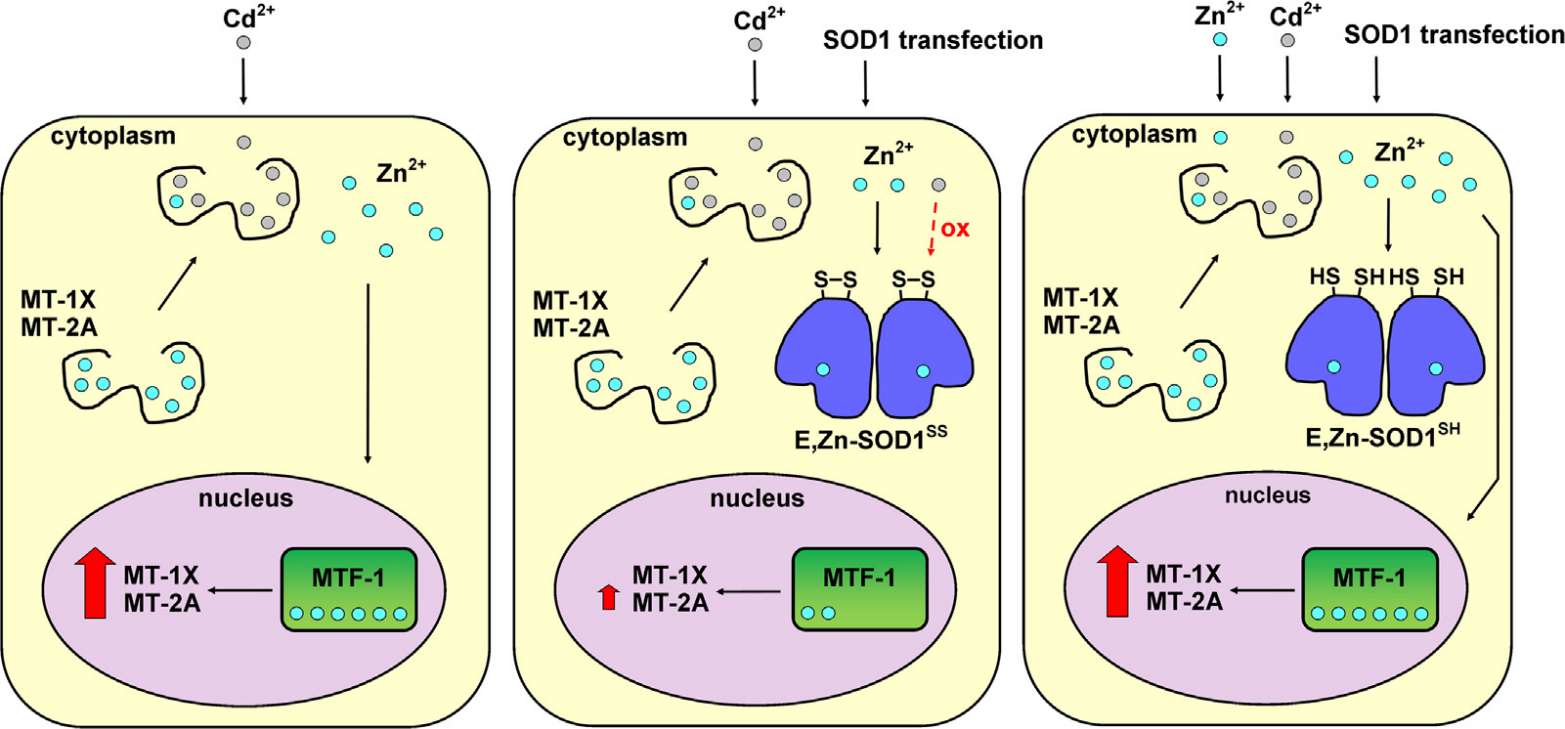
**Cd**^**2+**^**-induced SOD1 oxidation is modulated by Zn**^**2+**^**and MT levels.** Schematic drawing of the proposed mechanism of SOD1 oxidation as a function of Cd^2+^, Zn^2+^ and MTs. Left panel: when SOD1 is not overexpressed, Zn^2+^ (cyan) is displaced by Cd^2+^ (grey) and activates MTF-1 (green), which strongly induces MTs expression; the high levels of MTs contribute to restore the cellular redox balance by sequestering Cd^2+^. Centre panel: when SOD1 is overexpressed, it sequesters the Zn^2+^ displaced by Cd^2+^ causing a decreased MTs induction; Cd^2+^ is not efficiently sequestered by MTs and causes redox imbalance, leading to SOD1 oxidation. Right panel: when excess Zn^2+^ is supplemented, overexpressed SOD1 is metallated and the strong induction of MTs is restored, thus preventing SOD1 oxidation.

Such interplay between Zn^2+^, MT expression and SOD1 oxidation can be rationalised by considering the role of MTs in the cellular homeostasis of zinc. Zinc is an essential metal for the correct functionality and health of the cell. However, due to its high affinity for biological molecules, its intracellular availability has to be regulated to avoid uncontrolled binding to non-native acceptors. MTs, along with the other high affinity zinc-binding proteins, contribute to buffering the intracellular Zn^2+^ concentration [18]. Furthermore, both MTs and Zn^2+^ are involved in the cellular redox homeostasis [18], [56]. When intracellular Zn^2+^ increases, it binds to the metal regulatory transcription factor 1 (MTF-1), activating it and triggering the transcription of more MTs [21]. Consequently, when Cd^2+^ is added to the cells it displaces Zn^2+^ from MTs and other proteins, releasing it in the cytoplasm and causing it to activate MTF-1, inducing MTs overexpression (Fig. 5, left). In turn, overexpressed MTs protect the cells by directly binding Cd^2+^ and possibly by exerting other antioxidant functions [18]. When Cd^2+^ is added to cells overexpressing SOD1, the released Zn^2+^ is immediately sequestered by SOD1, which has a much higher affinity than MTF-1 and MTs, preventing the activation of MTF-1 and decreasing MTs overexpression as a consequence (Fig. 5, centre). Instead, when both Zn^2+^ and Cd^2+^ are supplemented, overexpressed SOD1 is fully metallated by the excess of Zn^2+^, so that the amount released by the MTs can still bind to MTF-1, restoring the high induction of MTs to NMR-detectable levels (Fig. 5, right). Finally, the different oxidation state of SOD1 observed when supplementing both Zn^2+^ and Cd^2+^ can be explained with the fact that in cells with higher levels of MTs the cellular redox balance is partially restored, preventing the premature oxidation of SOD1.

## 5 Conclusions

In this work, the effect of cadmium treatment on the metallation and redox state of SOD1 was investigated directly in human cells by in-cell NMR. Our study revealed an interesting relationship between the SOD1 redox state, the availability of Zn^2+^ and the Cd^2+^-induced overexpression of MTs. While Cd^2+^ itself does not bind SOD1 in place of Zn^2+^, it is able to induce the premature formation of SOD1 intramolecular disulfide bond, interfering with its correct maturation pathway. Furthermore, Cd^2+^ alters the cellular zinc homeostasis causing a strong induction of MTs. Such induction is negatively modulated by the overexpression of SOD1 itself, likely because the increased SOD1 levels interfere with the activation of the MT transcription factor MTF-1. While in our experiments the levels of SOD1 are artificially increased, our findings suggest that in cells with endogenously high levels of SOD1 (~40–100 µM), such as motor neurons and erythrocytes [57], [58], under certain conditions, SOD1 itself could sensitise the cells to Cd^2+^ toxicity, by sequestering the Zn^2+^ displaced by Cd^2+^, inhibiting the cellular response against Cd^2+^ and further altering the cellular redox balance.

Finally, as an interesting remark from the methodological point of view, we report that the strong induction of MTs resulted in the observation of NMR signals from MT-1X/MT-2A. To the best of our knowledge, this is the first time that signals from a protein synthesised by genomic DNA, *i.e.* not delivered into the cells nor ectopically expressed, could be detected by solution NMR in human cells.
