# Supplementary material for: Cadmium effects on superoxide dismutase 1 in human cells revealed by NMR
Source: Redox Biol. 2019 Jan 8;21:101102. doi: 10.1016/j.redox.2019.101102 (PMC6348768; doi:10.1016/j.redox.2019.101102)
Supplement: Supplementary file 1 — Supplementary material [file mmc1.docx]

Supporting Information

**Cadmium effects on superoxide dismutase 1 in human cells revealed by NMR**

Polykretis^a^, P., Cencetti, F.^b^, Donati, C. ^b^, Luchinat, E.^a,b,1^, Banci, L.^a,c,1^

^a^Magnetic Resonance Center - CERM, University of Florence, Via Luigi Sacconi 6, 50019 Sesto Fiorentino, Florence, Italy.

^b^Department of Experimental and Clinical Biomedical Sciences “Mario Serio”, University of Florence, Viale Morgagni 50, 50134 Florence, Italy.

^c^Department of Chemistry, University of Florence, Via della Lastruccia 3, 50019 Sesto Fiorentino, Florence, Italy.

^1^To whom correspondence should be addressed. Enrico Luchinat: telephone: +39 055 457 4245; e-mail: eluchinat@cerm.unifi.it. Lucia Banci: telephone: +39 055 457 4273; e-mail: banci@cerm.unifi.it.


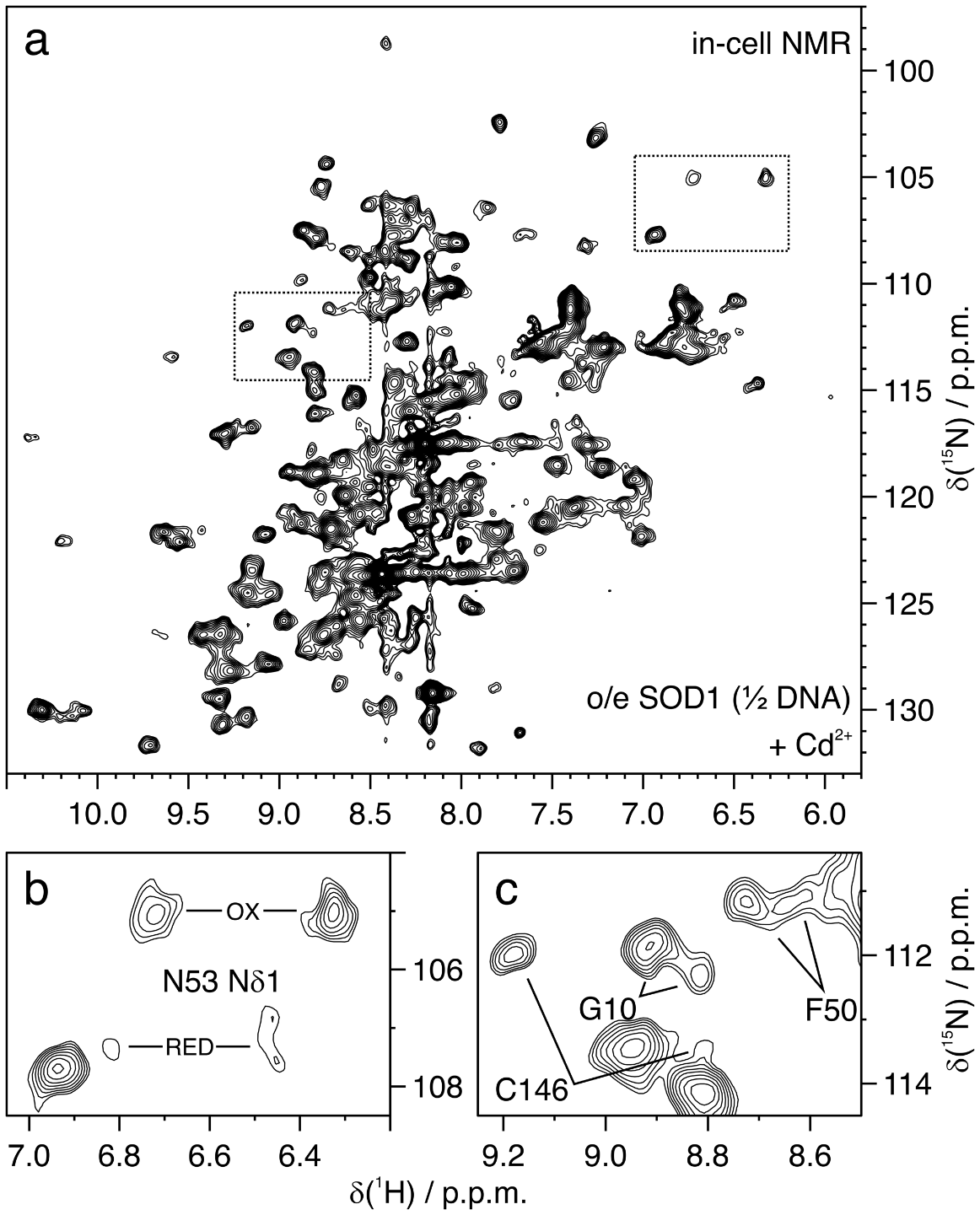


**Fig. S1. Cd^2+^ induces the oxidation of SOD1 expressed at lower levels.** (a) In-cell ^1^H-^15^N NMR spectrum acquired on cells transfected with a lower amount of SOD1 DNA, therefore expressing lower levels of [U-^15^N] labelled SOD1, in defect of Zn^2+^ and treated with Cd^2+^; (b, c) enlarged areas of the same spectrum (dotted rectangles in a). Representative signals affected by the formation of the C57-C146 disulfide bond are labelled.

**
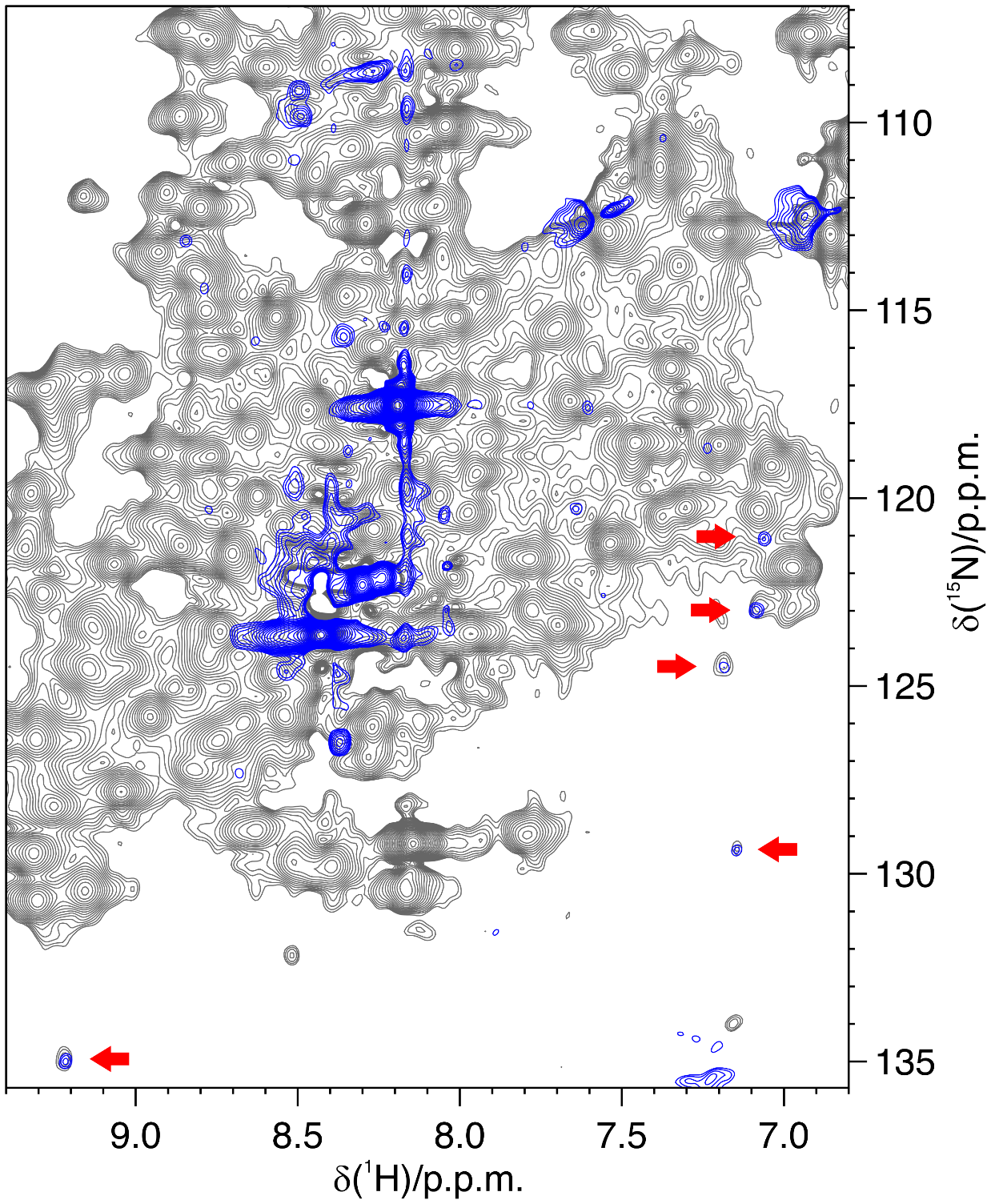
**

**Fig. S2. Cd^2+^-induced MTs are detected by NMR in cells overexpressing SOD1 in excess of Zn^2+^.** Overlay of ^1^H-^15^N NMR spectra acquired on human cells expressing [U-^15^N] labelled SOD1 in excess of Zn^2+^ and treated with Cd^2+^ (grey, same as the magenta spectrum in Fig. 3) and untransfected [U-^15^N] labelled cells (blue, same as the black spectrum in Fig. 4a). Signals arising from MTs that are not overlapped with SOD1 signals are indicated (red arrows).

**
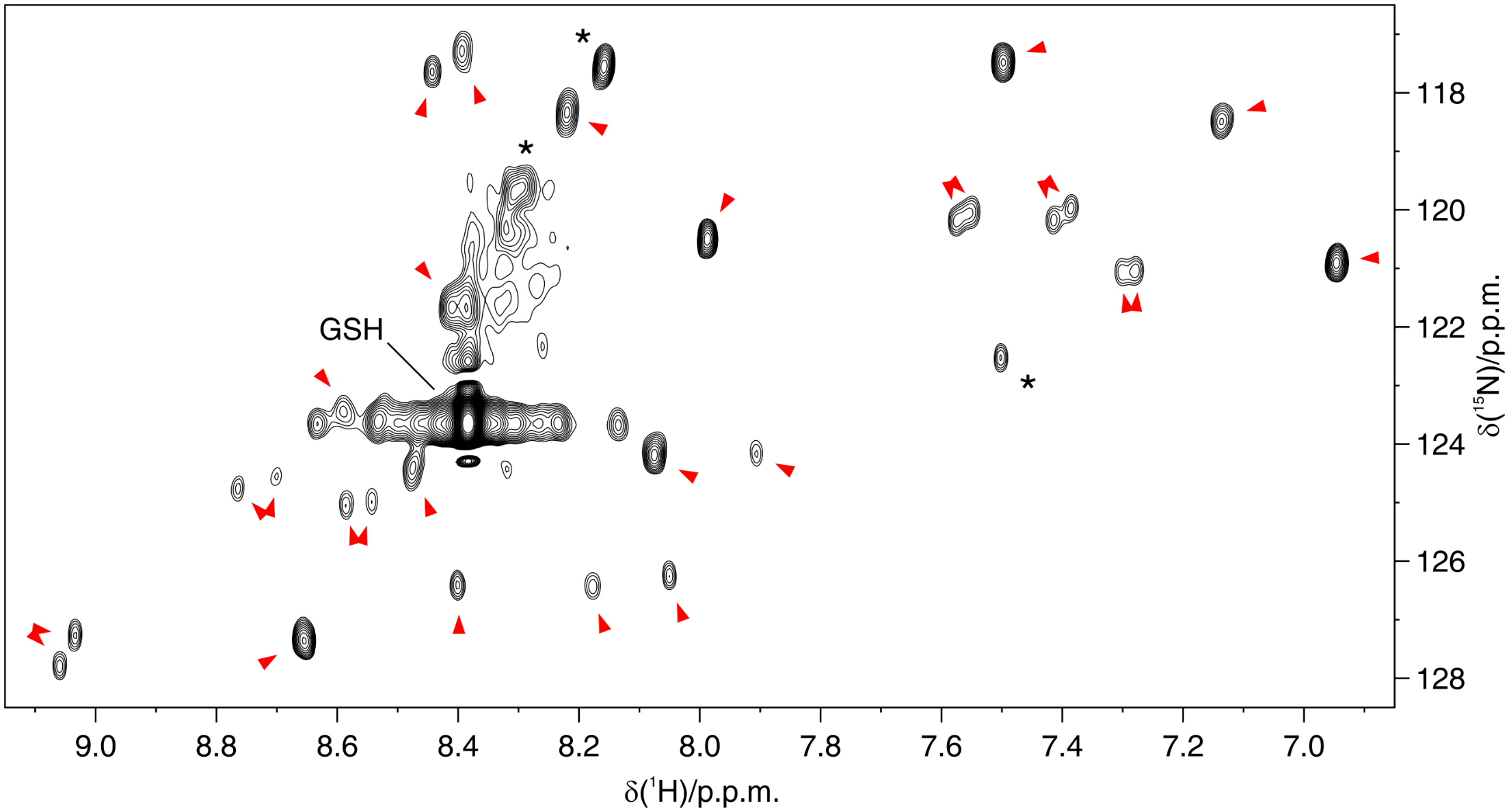
**

**Fig. S3. Many cysteine signals are detected following treatment with Cd^2+^.** ^1^H-^15^N NMR spectrum acquired for 18 hours at 298 K on the lysate obtained from untransfected, [^15^N]-cysteine labelled cells treated with Cd^2+^ (the corresponding in-cell NMR spectrum is shown in Fig. 4a, red). The strongest signal arises from [^15^N]-cysteine labelled glutathione (GSH). Signals attributed to overexpressed MTs are indicated with red triangles. Split signals that may arise from the same residue in different isoforms or in different metalation states are indicated with double triangles. Signals that were not attributed to MTs because they are also found in lysates from cells non-overexpressing MTs (data not shown) are labelled with an asterisk.


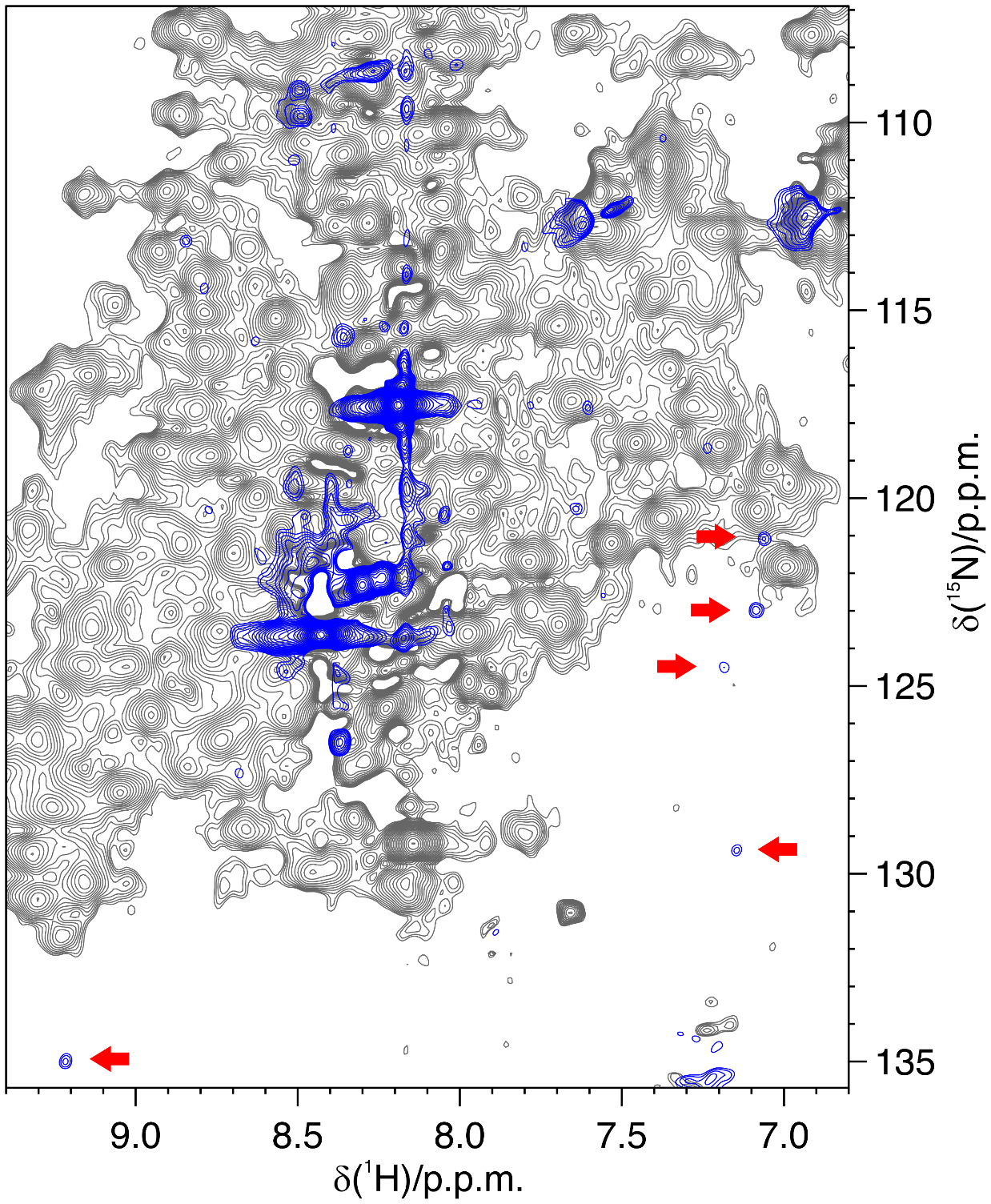


**Fig. S4. MTs are not detected by NMR in Cd^2+^-treated cells overexpressing SOD1 in defect of Zn^2+^.** Overlay of ^1^H-^15^N NMR spectra acquired on human cells expressing [U-^15^N] labelled SOD1 in defect of zinc and treated with Cd^2+^ (grey, same as the red spectrum in Fig. 1) and untransfected [U-^15^N] labelled cells (blue, same as the black spectrum in Fig. 4a). MT signals that would not overlap with SOD1 signals are indicated (red arrows).

**Table S1. TaqMan Gene Expression Assays employed for expression analysis of MTs.**

| **Gene** | **UniProtKB** | **Assay ID** |
| --- | --- | --- |
| MT1A | P04731 | Hs00831826_s1 |
| MT1B | P07438 | Hs01875377_s1 |
| MT1E | P04732 | Hs01938284_g1 |
| MT1F | P04733 | Hs00744661_sH |
| MT1G | P13640 | Hs04401199_s1 |
| MT1H | P80294 | Hs00823168_g1 |
| MT1M | Q8N339 | Hs00828387_g1 |
| MT1X | P80297 | Hs00745167_sH |
| MT2A | P02795 | Hs02379660_gH |

**Table S2. MTs expression in Cd^2+^-treated cells relative to basal levels and basal levels of each MT relative to MT1A. Values in 2^(-ΔΔCt) ± s.d. (n = 3)**

| **Gene** | **+ Cd^2+^ rel. to – Cd^2+^** | **– Cd^2+^ rel. to MT1A** |
| --- | --- | --- |
| MT1A | 1.1 ± 0.2 | 1 |
| MT1B | 1.26 ± 0.09 | 0.53 ± 0.04 |
| MT1E | 1.7 ± 0.1 | 1.25 ± 0.08 |
| MT1F | 21 ± 1 | 3.8 ± 0.2 |
| MT1G | 1.0 ± 0.1 | 0.96 ± 0.06 |
| MT1H | 2.70 ± 0.09 | 0.31 ± 0.04 |
| MT1M | n.d. | n.d. |
| MT1X | 276 ± 11 | 8 ± 1 |
| MT2A | 208 ± 12 | 60 ± 4 |

**Table S3. Expression of MT1X and MT2A in different conditions relative to basal. Values in 2^(-ΔΔCt) ± s.d. (n = 3)**

|  | **– Cd^2+^** | | | | **+ Cd^2+^** | | | |
| --- | --- | --- | --- | --- | --- | --- | --- | --- |
|  | **– o/eSOD1** | | **+ o/eSOD1** | | **– o/eSOD1** | | **+ o/eSOD1** | |
| **Gene** | **– Zn^2+^** | **+ Zn^2+^** | **– Zn^2+^** | **+ Zn^2+^** | **– Zn^2+^** | **+ Zn^2+^** | **– Zn^2+^** | **+ Zn^2+^** |
| MT1X | 1.00 ± 0.09 | 2.8 ± 0.4 | 1.1 ± 0.1 | 3.1 ± 0.3 | 205 ± 24 | 297 ± 29 | 92 ± 11 | 384 ± 72 |
| MT2A | 1.00 ± 0.07 | 1.8 ± 0.2 | 0.88 ± 0.09 | 1.64 ± 0.08 | 176 ± 35 | 259 ± 49 | 52 ± 7 | 175 ± 23 |
